# P63 and Ki-67 Expression in Dentigerous Cyst and Ameloblastomas

**Published:** 2015-12

**Authors:** Zohreh Jaafari-Ashkavandi, Bita Geramizadeh, Mohammad Ali Ranjbar

**Affiliations:** a Dept. of Oral and Maxillofacial Pathology, School of Dentistry, Shiraz University of Medical Sciences, Shiraz, Iran.; b Transplant Research Center, Dep. of Pathology, School of Medicine, Shiraz University of Medical Sciences, Shiraz, Iran.

**Keywords:** P63, Ki-67, Dentigerous Cyst, Ameloblastoma, Unicystic Ameloblastoma

## Abstract

**Statement of the Problem:**

P63 gene is a member of TP53 and its homologous gene family. Its expression was observed in some odontogenic lesions, more expression in aggressive lesions.

**Purpose:**

This study aimed to investigate the possible diagnostic impact of P63 protein on dentigerous cysts and various types of ameloblastoma. Its expression with Ki-67 proliferation marker was also compared.

**Materials and Method:**

This cross-sectional retrospective study was enrolled on 25 cases of dentigerous cyst including 21 unicystic ameloblastomas and 17 conventional ameloblastomas. The expression of P63 and Ki-67 was assessed by immunohistochemical (IHC) examinations. Data were analyzed by employing Mann-Whitney and correlation coefficient tests.

**Results:**

P63 expression was significantly higher in ameloblastoma than unicystic ameloblastoma and dentigerous cysts. There was no significant difference between unicystic ameloblastoma and dentigerous cyst in P63 expression. A 90% cut-off point was obtained for basal layer which gave 88% sensitivity and 78% specificity to distinguish more invasive lesions from others. There was not any correlation between P63 and Ki-67 immunostaining in the three study groups.

**Conclusion:**

More aggressiveness and more invasiveness of odontogenic lesions depicted higher rate and also more intensive expression of P63. Moreover, the expression of P63 protein had not any correlation with Ki-67 protein in dentigerous cysts and ameloblastomas.

## Introduction

Odontogenic cysts and tumours arise from the odontogenic epithelium of tooth germ. Ameloblastoma is the most common odontogenic tumour with clinical significance. This neoplasm appears in three forms: conventional, unicystic and peripheral. These lesions present different clinical and histopathological features which need different managements. Conventional type is a locally invasive benign tumour with high recurrence rate.[1-2] Unicystic ameloblastoma mimics the dentigerous cyst in clinical, radiographical, and even histopathological features. Unicystic ameloblastoma may arise from a dentigerous cyst, but these odontogenic lesions have different clinical behaviours and treatment managements. Therefore, accurate diagnosis and identifying the processes which explain the tumour growth and invasion are the matter of concern.

TP63 (p63) is a homologue of TP53 gene and is located at the 3q27-29 locus. P63 has two promoters and produces two types of protein: TAP63 that contains acidic N-terminal trans-activation domain and ∆NP63 that lacks this domain.[3-4] Expression of P63 protein is discovered in skin, oesophagus, oral mucosa, prostate, breast, lung, salivary glands, and odontogenic epithelium of tooth germ as well as dental follicle of impacted teeth.[5-7] Studies showed that P63 is an essential protein for epithelial stratification[8] and various isoforms of P63 have different roles. ∆NP63 proteins contribute to cell proliferation; while, TAP63 isoforms induce cell differentiation.[9]

There are some researches on expression of P63 in odontogenic cysts and tumours.[10-14] They reported that more aggressive tumours such as keratocystic odontogenic tumour (KCOT) and ameloblastoma have more expression of P63.[10-12] However, most of these studies demonstrated semi-quantitative data and did not present any cut-off point to help diagnosis. Ameloblastomas, unicystic and solid types, may arise from a dentigerous cyst and may show transitional changes from a non-aggressive cyst to a locally invasive tumour. Therefore, we analyzed the comparative expression of P63 in these lesions to evaluate this protein as a marker in early diagnosis which consequently helps selecting accurate management. Ki-67 is the most frequently applied proliferation marker for evaluating proliferative activity and biologic behaviour of many pathologic lesions, including odontogenic cysts and tumours.[15-16] Regarding the role of P63 protein in epithelial cell proliferation, we also evaluated the correlation of Ki-67 and P63 in those odontogenic lesions.

## Materials and Method

In this retrospective cross-sectional analytical study, 25 cases of dentigerous cyst including 21 cases of unicystic ameloblastoma and 17 ameloblastomas were selected from the archive of oral pathology department, School of Dentistry, Shiraz University of Medical Sciences. The samples had adequate epithelial component to evaluate the quantity of stained cells. Clinical data including lesion site, age and sex of patients were obtained from patients’ registered medical documents. Cases with uncertain diagnosis, severe inflammation, and small size lesions were excluded.

**Immunohistochemistry**

After reviewing and confirming the proposed diagnosis of tumours, two 4µm sections of formalin-fixed and paraffin-embedded specimens were deparaffinised in xylene, then rehydrated by using various concentrations of alcohol, and finally washed with distilled water. Immunohistochemistry (IHC) was performed by using envision-labelled peroxidase system (DAKO Carpinteria; CA, USA). Antigen retrieval was performed by DAKO Cytomation target retrieval solution (PH=9) for 20 minutes. Endogenous peroxidase activity was blocked by 0.3% H2O2. Antigen-antibody reaction was detected by mouse monoclonal anti-P63 antibody (clone 7JUL; Ready to Use, Novocastra, Newcastle, UK) for one hour at room temperature. Then, 3, 3 di-aminobenzidine (DAB Liquid K3467; DAKO Corporation, Denmark) was used as chromogen. Sections were counterstained with Harris' Hematoxylin, washed with tap water and covered by glass coverslips. The positive control was normal oral mucosa for both antibodies. As a negative control, primary antibody was replaced by phosphate-buffered solution. The mean percentage of P63- and Ki-67- positive cells was calculated in 200 cells selected from at least three random fields (at 400x magnification). In dentigerous cysts and luminal unicystic ameloblastoma, brown nuclei in basal layer and suprabasal layers (100 cells for each) and in mural and conventional ameloblastomas, peripheral and central cells of ameloblastic islands (100 cells for each) were considered to be scrutinized. Intensity of P63-staining was also evaluated in three groups: I (mild, light- brown), II (moderate), III (severe, dark-brown).

**Statistical analysis**

Data was analysed by using SPSS software (version 11). T-test, Mann-Witney, and ‍‍‍correlation tests were used as appropriated. P-value<0.05 was considered as significant. A receiver operating characteristic (ROC) curve was obtained to distinguish more invasive lesions (mural and solid ameloblastoma) from other non-aggressive lesions. 

## Results

The patients were 33 men and 30 women with the mean±SD age of 27±15.2 years. The brown nuclei were indicative of P63 and Ki-67 expression.

**P63 expression**

**Figure 1:**
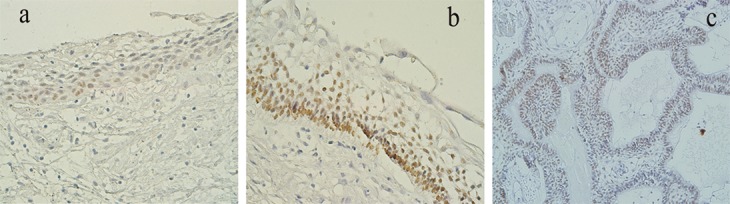
a: P63 expression in dentigerous cyst in only basal layer.  b, c: P63 expression in luminal (b) and mural (c) unicystic ameloblastoma. Intense immunostaining in lower layers (×400 magnification)

In dentigerous cyst group; P63- positive cells were mainly located in basal cell layer of epithelial lining and the layers above this area had a lower mean of expression (Figure 1a); however, Mann-Whitney test showed that this difference was not statistically significant (*p*= 0.3). Superficial cells did not show any staining. The mean of P63 expression and its intensity are summarized in tables 1 and 2. There were 5 cases with mild to moderate inflammation that showed a slightly lower P63-expression, but it was not significantly different with non-inflamed cysts (*p*> 0.05).

**Table 1:** P63 expression (mean±SD) in various cell layers and Ki-67 Labelling Index (LI) in the three study groups

	**P63 expression**			**Ki-67 LI**
	**Basal/Peripheral cells**		**Suprabasal/Central cells**	
Dentigerous Cyst (n=25)	71.8 ± 23.1		55.8± 25.8	2.4± 2.3
Unicystic Ameloblastoma(n=21)	75.8± 9.9		71.5± 15.9	2.9± 2.5
Luminal(4)	71.25± 32.3		64.7± 14	-
Mural(17)	77 ± 30.2		73.2 ± 16.3	-
Ameloblastoma(n=17)		97.7 ± 5	80.3± 16.6	5.4± 4.4

**Table 2:** Intensity of P63 expression in study groups

	**Mild** **N (%)**	**Moderate** **N (%)**	**Severe** **N (%)**	**Total**
Dentigerous cyst	(40) 10	(52) 13	2(8)	(100) 25
Unicystic A.	(9.5) 2	(52.4) 11	(38.1) 8	(100) 21
Ameloblastoma	(5.9) 1	(29.4) 5	11 (64.7)	17(100)
Total	(20.6)13	(46) 29	(33.3) 21	63(100)

A: Ameloblastoma

**Figure 2:**
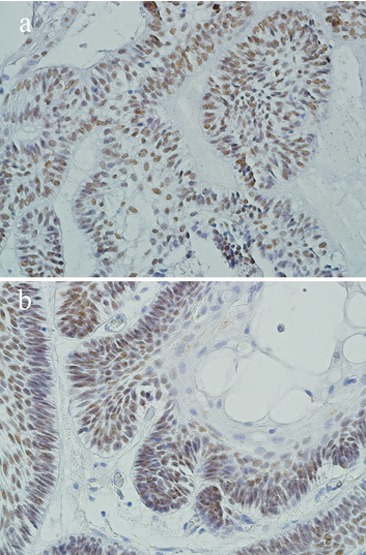
a: P63 expression in ameloblastoma (×400 magnification). b: Squamous metaplasia does not show P63 expression.

The unicystic ameloblastomas consisted of 4 cases of luminal and 17 cases of mural types. The pattern of P63 expression was similar to dentigerous cysts. Basal layer showed more positive cells; however, the intensity of staining was higher (Table 2, Figure 1b, c). There was no significant difference between the cystic lining of luminal and mural ameloblastomas (*p*= 0.38 by Mann-Whitney test). The ameloblastoma group included 10 follicular and 7 plexiform subtypes. P63 positive cells were found in most peripheral and central cells of ameloblastic nests and in both histological subtypes (Table 1, Figure 2a). However, squamous metaplasia and lining of microcysts were not labeled (Figure 2b). Follicular and plexiform ameloblastomas did not show significant difference in P63 expression.

There was no significant difference in overall P63 expression between dentigerous cyst and unicystic ameloblastoma; however, T-test analysis showed a statistically significant difference between P63 expression in dentigerous cyst and luminal unicystic ameloblastoma in suprabasal (*p*= 0.02), but not in basal layers (*p*= 0.5). Also, peripheral cells of ameloblastic nests in solid and mural ameloblastomas revealed a significant difference in labelling P63 (*p*= 0.01), but not in central cells.

Overall P63 expression was significantly higher in ameloblastoma than unicystic ameloblastoma and dentigerous cyst according to t-test (*p*= 0.04 and 0.002, respectively). The intensity of staining was evaluated by using Chi-square test (Table 2) and the results showed that intensity was significantly different between the three groups (*p*= 0.001); however, there was no difference between various tumour subtypes.

**Ki-67 expression**

Ki-67-positive staining was detected in 40 cases including 13 cases of dentigerous cyst, 13 cases of unicystic ameloblastomas, and 14 cases of solid ameloblastoma. Ki-67 was labelled heterogeneously in basal and suprabasal layers of cystic samples and in peripheral and central cells of ameloblastic nests. Ki-67-labelling index (LI) was 2.4±2.3 in dentigerous cysts, 2.9±2.5 in unicystic, and 5.2±4.4 in solid ameloblastoma (Table 1). Solid ameloblastoma had significantly higher LI than unicystic ameloblastoma and dentigerous cyst. The difference in Ki-67 LI between dentigerous cyst and unicystic ameloblastomas was not statistically significant. The correlation test revealed no correlation between P63 and Ki-67 expression in the study groups.

**Figure 3:**
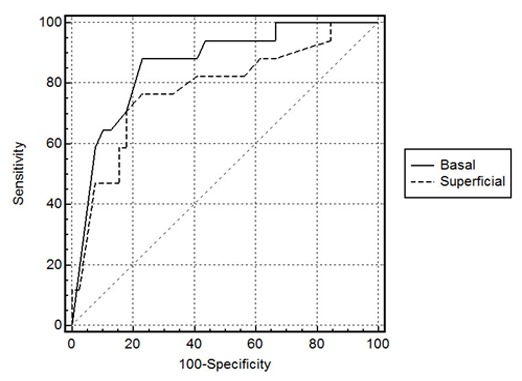
ROC curve for basal and superficial layers: evaluation of basal layer is better for differentiation of aggressive lesions

According to the receiver operating characteristic (ROC) curve analysis, we obtained a 90% cut-off point for basal layer which gave 88% sensitivity and 78% specificity to distinguish more invasive lesions including mural and solid ameloblast from others (dentigerous and luminal). The area under ROC curve was 0.84 with 95% confidence interval (CI). Cut-off point for suprabasal layers was 77% with 72% sensitivity and 77% specificity. The area under this curve was 0.75. This analysis demonstrated that basal layer was a better area than suprabasal layers to differentiate aggressiveness of the lesions (Figure 3).

## Discussion

In this study, the expression of P63 protein was evaluated since previous studies reported its effects on aggressive behaviour of odontogenic cysts and tumours. We also assessed the correlation of P63 expression with Ki-67 proliferation marker. The expression of P63 with mild intensity was found in basal and parabasal layers of cystic samples, but not in superficial layers. Earlier studies surveyed P63 protein in odontogenic cysts and reported that staining was not observed in superficial layers of the cysts other than odontogenic keratocyst (currently known as keratocystic odontogenic tumour, KCOT) and that non-aggressive cysts showed mild expression of P63.[10-11] Immunoreactivity for this protein in luminal lining of unicystic ameloblastoma was similar to that in dentigerous cyst; however, the intensity of staining was higher and suprabasal layers showed greater percentage of stained cells. Unicystic ameloblastoma in many cases shows transitional changes from dentigerous cyst to an ameloblastoma. Moreover, some researchers demonstrated that in the epithelial linings of KCOT, unlike dentigerous and radicular cysts, P63-positive cells were present from basal to upper cell layers with more intensity.[10, 12-13] Therefore, pattern and intensity of P63-staining might be a diagnostic aid for more aggressive cysts.

In the present study, the immune reaction of P63 was slightly lower than that in inflamed cysts, though not statistically significant. Gonçalves *et al.* found this finding in the cases of severe inflamed radicular cyst.[12]Our study excluded severe inflamed cysts.

In a similar way, the infiltrating nests in mural ameloblastoma and solid type showed severe staining in most of the peripheral and many central cells. Mural and solid ameloblastomas are locally infiltrative neoplasms which need a more invasive surgical treatment than dentigerous cyst and luminal unicystic ameloblastoma. Moreover, we analyzed this marker as a diagnostic aid to distinguish aggressive from non-aggressive odontogenic lesions which have clinicopathological similarities. According to the results, 90% or more staining in the basal layer supported mural and solid ameloblastomas, which should be considered for more extensive surgical management and longer follow-up than non-aggressive cystic lesions. Our results showed that evaluation of basal layer was more accurate than suprabasal layers for differentiation of these lesions. These results may be useful in small biopsied specimens in which the final diagnosis is not simple. Our results support the hypothesis which construed that P63 protein may contribute to the tumour genesis of odontogenic structures.[13]

In the present study, less differentiated cells that were located in basal cell layer of cystic lesions and in the tumoral nests displayed extensive P63- immunoreactivity; whereas, terminal differentiated cells like squamous cells and the lining of microcysts did not show staining. It seems that during the transformation of a cyst to a tumour, the upper cell layers lose their differentiation and express P63. This figure was also reported by Kumamoto *et al.* in keratinized and granular cells of ameloblastoma.[6] These features support anti-differen-tiation activity P63 in odontogenic cyst and tumours.

Some authors stated that P63 was in association with epithelial cell proliferation due to the expression pattern of this protein in basal and parabasal layers in epithelial component of mucosa and cysts.[10-11,17-18]In the present study, we analysed Ki-67 proliferation marker in the samples and evaluated its LI in comparison with P63-expression. Ki67-positive cells were found in basal and parabasal layers of cystic lesions, and peripheral and central cells of ameloblastomas. Ki-67 LI did not show any significant different between dentigerous cyst and unicystic ameloblastoma. It may be attributed to the slow growth of unicystic ameloblastoma and its lower aggressive behaviour in comparison with solid ameloblastoma. According to the results of the present and previous studies, P63 protein is expressed in proliferative compartment of odontogenic lesions.[10-11]Nevertheless, our statistical analysis did not show any correlation between expression of Ki-67 and P63 markers. In contrast with these results, Vered *et al.* found a correlation between Ki-67 and P63 immunoreaction in epithelial dysplasia and oral squamous cell carcinoma.[19] In agreement with our findings, Takada *et al.* have found an increasing Ki-67 LI with progression of dysplasia, but P63 expression has not risen. They included that P63-positive cells could provide stem cell features rather than direct correlation with carcinogenesis.[20]Because of the presence of P63-positive cells in the suprabasal layers of cystic lining and central cells in ameloblastic nests, this reaction probably was indicator of amplifying cells in addition to the stem cells. Takeda *et al.* indicated that only intense-stained P63 positive cells were true stem cells.[20] P63 expression was also displayed in basal and parabasal cell layers of oral epithelium and epidermis, where the amplifying or transient amplifying cells were found.[21-22] Also, it seemed that other stem cell markers such as CK19 did not have any particular relation with P63-positive cells.[20] P63 positive cells are necessary for proliferation; however, their presence is not indicator of proliferation and anti-differentiation activity is among their important roles, as well.

Various P63 isoforms have different functions and the balance between these isoforms varies during formation and progression of the tumour. Therefore, to determine the major role of P63 in tumour genesis of a given tumour, the type of dominant isoform is important.[23] Therefore, PCR technique was required to determine the dominant type.

## Conclusion

P63 and Ki-67 had higher expression in more aggressive ameloblastic lesions. Therefore, over expression of P63 and Ki-67 in combination with histomorphological examination may provide useful diagnostic aid for aggressive odontogenic epithelial cysts and tumours, with a 90% cut-off point for P63 staining in basal layer (88% sensitivity and 78% specificity). Also, evaluation of basal layer was more precise than suprabasal layers. P63 positive cells were present in basal and suprabasal layers. The pattern of immunoreactions seemed to be related to anti-differentiation and proliferation activity of this protein. We suggest further studies to assess various isoforms of P63 in the odontogenic lesions and their correlation with Ki-67 positive cells. 
